# Unraveling the hidden paleobiodiversity of the Middle Devonian (Emsian) crinoids (Crinoidea, Echinodermata) from Poland

**DOI:** 10.7717/peerj.12842

**Published:** 2022-02-09

**Authors:** William I. Ausich, Mariusz A. Salamon, Bartosz J. Płachno, Tomasz Brachaniec, Wojciech Krawczyński, Andrzej Boczarowski, Karolina Paszcza, Magdalena Łukowiak, Przemysław Gorzelak

**Affiliations:** 1 University of Columbus, Columbus, OH, USA; 2 University of Silesia in Katowice, Sosnowiec, Poland; 3 Jagiellonian University in Kraków, Kraków, Poland; 4 Insitute of Paleobiology, Warszawa, Poland; 5 Insitute of Paleobiology Polish Academy of Science, Warszawa, Poland

**Keywords:** Devonian, Emsian, Poland, Crinoids, Echinodermata, Diversity

## Abstract

Most previous publications on Devonian crinoids from the Holy Cross Mountains in Poland have concentrated on crinoid columns, and until now, little has been published about crinoid cups and calyxes. Herein, five crinoid taxa are described from an abundant occurrence of aboral cups and partial crowns from the Bukowa Góra Member (Emsian) in the Holy Cross Mountains of southern Poland. The following taxa are described: *Bactrocrinites* sp., *Codiacrinus sevastopuloi* sp. nov., *Halocrinites geminatus* (Bohatý, 2005), *Halocrinites schlotheimii* Steininger, 1831, and a single brachial plate from a flexible crinoid placed in Flexibilia *incertae sedis*. Simple discoid holdfasts are also present encrusted to cylindrical stromatoporoids. These taxa are the first crinoids described from the remains of partial crowns from Emsian strata of Poland.

## Introduction

**Figure 1:**
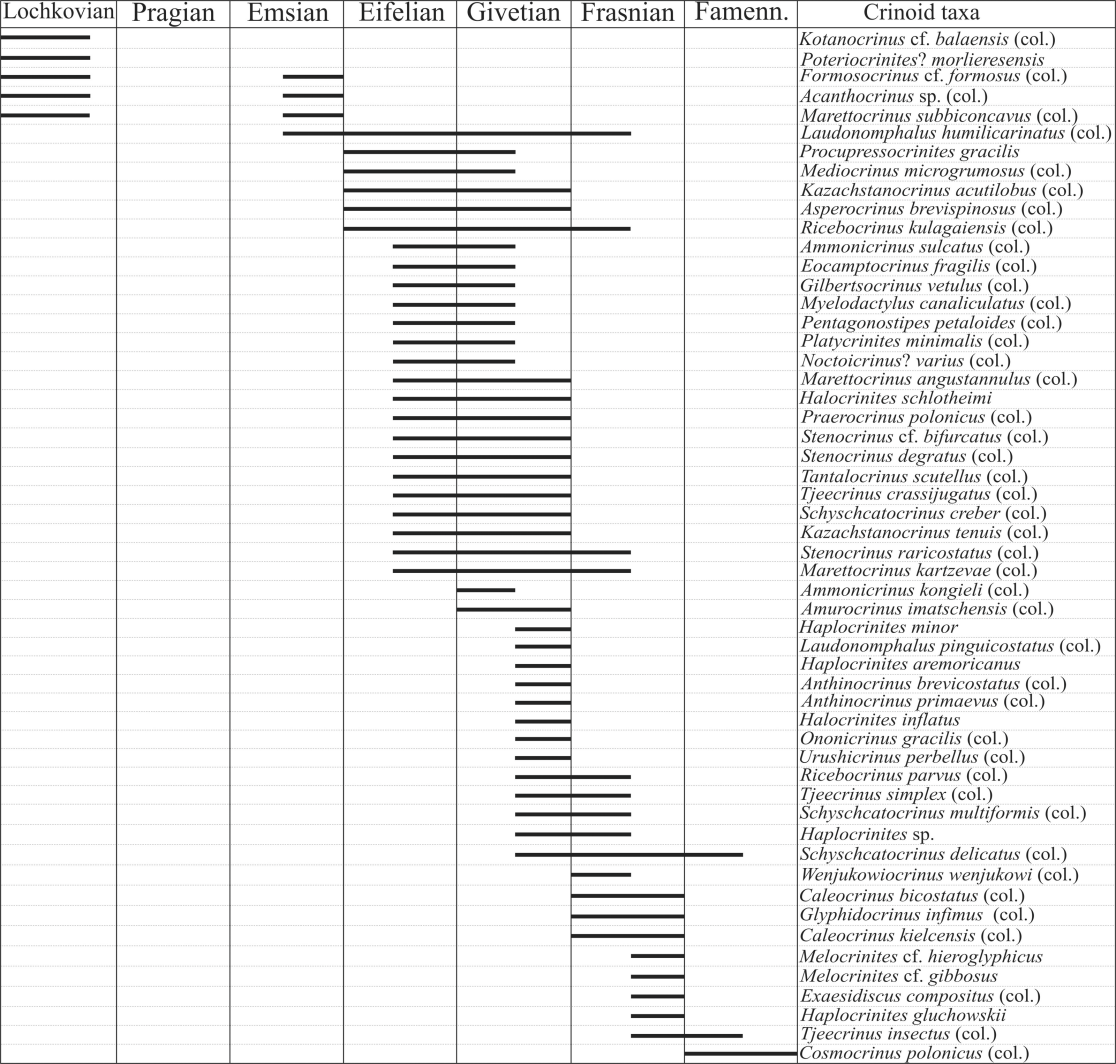
Stratigraphic ranges of crinoid taxa recorded in Devonian of Poland. Data compiled after: Dames (1868), Zeuschner (1867), Zeuschner (1869), Gürich (1896), Sobolev (1909), Kongiel (1958), Piotrowski (1977), Głuchowski (1980), Głuchowski (1981a)–Głuchowski (1981c), Głuchowski (1993a), Głuchowski (1993b), Głuchowski (2002), Głuchowski (2003) and Hauser (2002).

Crinoid remains are abundant in Devonian (Emsian-Famennian) strata of Poland (Holy Cross Mountains, southern Poland; Cracow-Silesian area, southern Poland; Sudetes, southwestern Poland; Pomerania, northern Poland). Polish Devonian crinoids were mentioned initially by Dames (1868), Zeuschner (1867), Zeuschner (1869), Gürich (1896), and Sobolev (1909). Much later, Kongiel (1958) and Piotrowski (1977) described the occurrence of the genus *Ammonicrinus* in the Holy Cross Mountains (see also Gorzelak, Głuchowski & Salamon, 2014; Bohatý, 2011). In a series of subsequent papers, Gluchowski (Głuchowski, 1980, Głuchowski, 1981a, Głuchowski, 1981b Głuchowski, 1981c, Głuchowski, 1993a, Głuchowski, 1993b, Głuchowski, 2002, Głuchowski, 2003; see also Hauser, 2002, Bohatý, 2005, Bohatý, 2009a, Bohatý, 2011) listed ∼50 crinoid taxa from Devonian (Lochkovian, Emsian-Famennian) strata of Poland (for summary see Fig. 1). With the exception of taxa listed below, most of them were based on isolated skeletal remains, mainly columnals; and they were described using the principles of artificial classification of crinoid remains proposed by Moore & Jeffords (1968). Głuchowski (2003) added that the applicability of crinoid stems may be useful for stratigraphic and correlation purposes.

To date, only nine crinoid taxa have been identified on the basis of complete (or nearly complete) crowns and aboral cups with column from the Devonian of Poland. Among these is *Haplocrinites* sp. from Givetian-Frasnian of Holy Cross Mountains recorded by Głuchowski (1993a) and Głuchowski (2003). Specimens of this taxon from late Frasnian Detrital Beds of the Holy Cross Mountains were later designated by Hauser (2002) as *Haplocrinites gluchowskii* (Hauser, 2002). According to Głuchowski, Casier & Olempska (2006) Givetian and early Frasnian *Haplocrinites* sp. specimens differ from *H*. *gluchowskii* in having distinctly less prominent radial facets. Another haplocrinitid (Haplocrinitidae) species is *Haplocrinites aremoricensis* Le Menn, 1985 from the uppermost Givetian of the Holy Cross Mountains (Głuchowski, 1993a). *Platyhexacrinus*? was identified by Głuchowski (1993a) (see Bohatý, 2009a). Also four cupressocrinitid (Cupressocrinitidae) taxa have been described from the Holy Cross Mountains by Głuchowski (1993a). These are *Cupressocrinites* cf. *abbreviatus* Goldfuss (late Eifelian-late Givetian) (now *Halocrinites schlotheimii* Steininger, 1831), *H. geminatus* (Bohatý, 2005) (these specimens were originally described as *Cupressocrinites* cf. *abbreviatus* in (Głuchowski, 1993a, fig. 6g–h), *C*. *inflatus* (Schultze, 1866) (late Givetian) (now *Halocrinites inflatus*), and *C. sampelayoi* (Almela & Revilla, 1950) (now *Halocrinites minor* (Schultze, 1866) known from late Givetian (Głuchowski, 1993a; Głuchowski, 2002). The remaining two crinoid species belong to the Melocrinitidae. These are *Melocrinites* cf. *gibbosus* Goldfuss, 1831 and *M*. cf. *hieroglyphicus* Goldfuss, 1831, which were found in the sediments of the uppermost Frasnian.

Only four crinoid taxa are known from the Emsian of the Holy Cross Mountains and all were documented on the basis of isolated columnals or their impressions (casts). In particular, Głuchowski, (1981b), Głuchowski, (2002) listed the following taxa from the Bukowa Góra shales: *Acanthocrinus* sp. (col.) and *Formosocrinus* cf. *formosus* (col.) (Yeltyschewa & Sisova, 1973), *Laudonomphalus humilicarinatus* (col.) (Yeltyscheva in Yeltyschewa & Dubalotova, 1961) [now *Hexacrinites*? *humilicarinatus* (col.) (note that an affiliation with the crown-based genus, *Hexacrinites*, cannot be verified.)], and *Marettocrinus subbiconcavus* (col.) (Stukalina, 1965).

Here we report complete or almost complete Emsian cups associated with numerous isolated calyx and column remains from the Bukowa Góra Member in the Holy Cross Mountains of southern Poland. These include *Bactrocrinites* sp., *Codiacrinus sevastopuloi* sp. nov., *Halocrinites geminatus* (Bohatý, 2005), *Halocrinites schlotheimii* (Steininger, 1831), and Flexibilia incertae sedis. Simple discoid holdfasts are also present. Remains of unidentifiable specimens indicate that several other crinoids also existed in the Bukowa Góra Member fauna.

## Geologic Framework

**Figure 2:**
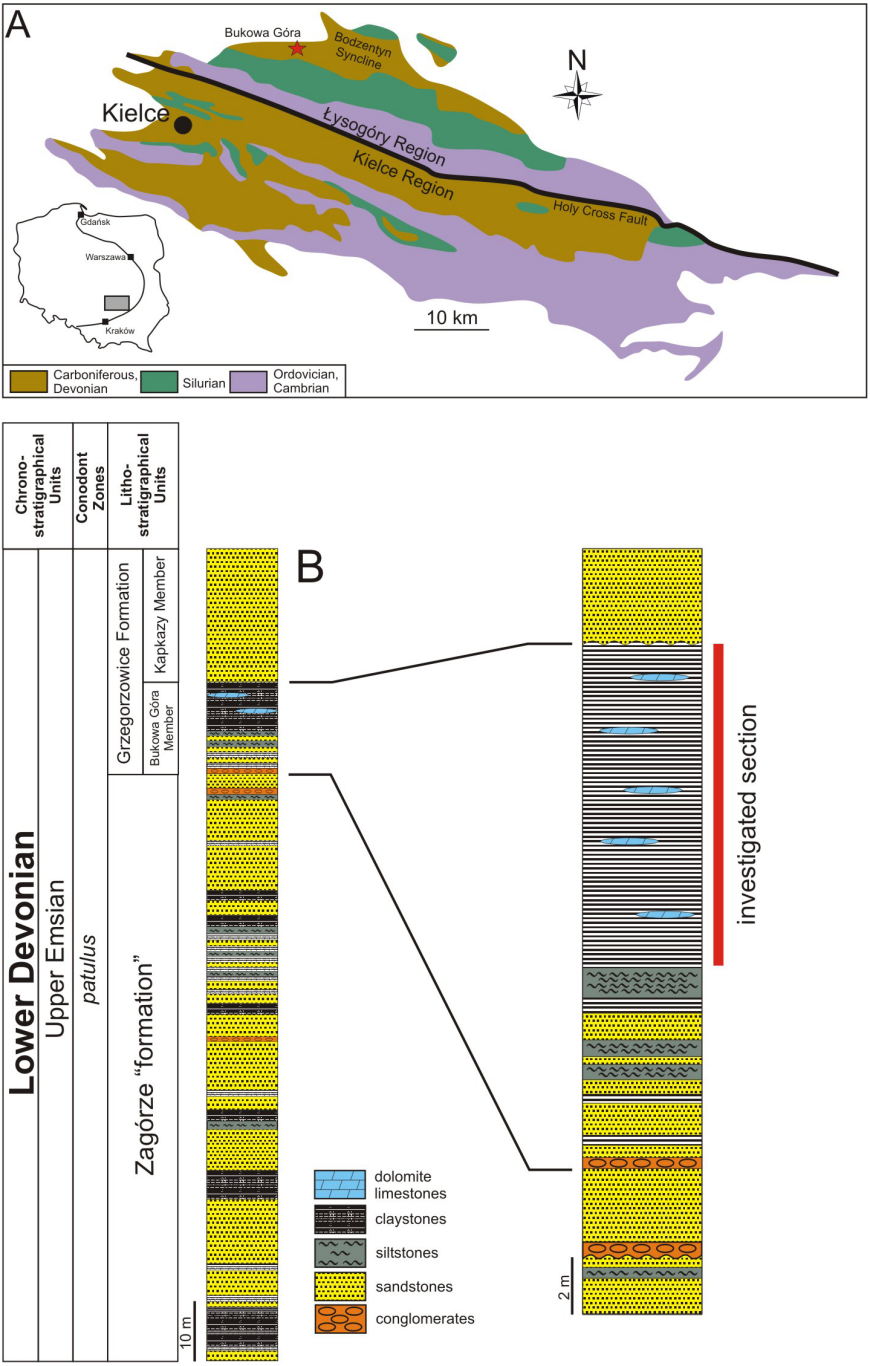
(A) The map of Poland with the Holy Cross Mountains area marked as grey rectangle. (B) The lithostratigraphical scheme of Middle and Upper Devonian in the Bukowa Góra Quarry. Compiled after Marynowski, Salamon & Narkiewicz (2002), Malec (2005), Szulczewski & Porȩbski (2008), Wójcik (2015), and Salamon et al. (2018).

The Holy Cross Mountains are located in the southern part of Poland. Their main element is the Paleozoic core, divided into two parts: the Łysogóry region (northern, connected with the Łysogóry Block) and the Kielce region (southern, connected with the Małopolska Block; see Fig. 2A). These regions differ from each other by facies development of contemporaneous deposits. Devonian sediments of the Łysogóry region were formed in the deeper basin in contrast to the shallower facies exposed in the Kielce region (Szulczewski, 1995).

Outcrops of Lower Devonian rocks in the Łysogóry Region are connected with the southern limb of the Bodzentyn Syncline. The sedimentary rocks of the upper Emsian are best exposed in the active quarry “Bukowa Góra”, located about 16 km northeast of Kielce (see Fig. 2A). The section includes sediments belonging to *patulus* Conodont Zone and *douglastownense-eurypterota* Miospore Zone Malec, 2005; Filipiak, 2011; Fijałkowska-Mader & Malec, 2011).

In the lower part of the section, the 110 m thick Zagórze “formation” comprised of siliciclastic deposits is present (see Fig. 2B). They are mostly represented by quartzitic sandstones with abundant trace fossils and by claystones. Within sandstones, there are storm originated brachiopod coquinas with gastropods, bivalves, tentaculitids, crinoids, rarely trilobites, rugose corals, nautiloids, and ostracodes. At the top of the Zagórze “formation”, conglomerates and sandstones of estuary facies crop out. Deposits of the Zagórze “formation” were formed in the shallow sea environment from the lagoon to the shoreface (Szulczewski & Porębski, 2008; see also Łobanowski, 1971, Łobanowski, 1981).

As a result of the progressive deepening of the marine basin, deposits of the Grzegorzowice Formation were formed, which is also present in the Kielce region (Malec, 2005; Wójcik, 2015). In the Bukowa Góra quarry section the two members are present: Bukowa Góra Member and the Kapkazy Member. The Bukowa Góra Member occurs only within the *patulus* Conodont Zone in the western part of Bodzentyn Syncline (see Fig. 2B). In the eastern part of the Bodzentyn Syncline, the Bukowa Góra Member appears earlier, *i.e.*, in the *serotinus* Conodont Zone (Malec, 2005). Malec (2005) marked the lower boundary of the Bukowa Góra Member in the bottom of the complex of dark claystones, whereas Szulczewski & Porębski (2008) put this boundary on the pebble conglomerate that begins in the lower shoreface to offshore transitional series.

In the lower part of interbedded sandstones, the Bukowa Góra Member is comprised of sandstones, siltstones, and claystones, which are about 7 m thick (see Szulczewski & Porębski, 2008). Above them appears the offshore facies represented by black to dark-gray claystones and silty claystones with a thickness of ∼13 m and containing discontinuous beds of dolomitic limestones up to 10 cm thick (see Fig. 2B). Both claystones and limestones contain a rich faunal assemblage related to the colonization of the soft sea bottom. There are massive colonies of both stromatoporoids and tabulate corals accompanied by solitary rugoses, brachiopods, crinoids, ostracods, gastropods, and trilobites (Malec, 2005; see also Głuchowski, 1993b; Fijałkowska-Mader et al., 1997).

Claystones of the Bukowa Góra Member are overlain by sandstones of the Kapkazy Member, which is ∼34 m thick. The lower part of the Kapkazy Member is comprised of coarse-grained and conglomeratic sandstones, containing rare crinoids, brachiopods, and gastropods. Above this is fine-grained sandstone, which is indicative of a clear shallowing of the sea basin (Malec, 2005).

## Materials and Methods

The studied material from Bukowa Góra Quarry was collected in 2019 and 2021. The first step consisted of examination of slab surfaces in the field. At this stage, numerous crinoid remains (isolated columnals and complete or nearly complete crowns) were collected. The next step consisted of soaking the respective samples (11 shales samples weighing each ca. 10 kg) only with hot water. Limy samples (4 samples weighing each ca. 5 kg) were soaked with Glauber’s salt. These samples were then boiled and frozen (2–3 times). The residues were finally washed with running tap water and sieved on a sieve column (Ø1.0, 0.315 and 0.1 mm mesh). The final step consisted of drying the shaly and limy residues at 160 ° C. Residue was hand-picked from each macerated sample for microscopic study.

All crinoids were photographed by a SONY DSC-RX10M3 digital camera. Specimens discussed here are deposited in the University of Silesia in Katowice, Faculty of Natural Sciences, Institute of Earth Sciences, Poland (GIUS 4-3696) and in the Senckenberg Forschungsinstitute und Naturmuseum, Frankfurt am Main, Germany (SMF).

“The electronic version of this article in Portable Document Format (PDF) will represent a published work according to the International Commission on Zoological Nomenclature (ICZN), and hence the new names contained in the electronic version are effectively published under that Code from the electronic edition alone. This published work and the nomenclatural acts it contains have been registered in ZooBank, the online registration system for the ICZN. The ZooBank LSIDs (Life Science Identifiers) can be resolved and the associated information viewed through any standard web browser by appending the LSID to the prefix http://zoobank.org/. The LSID for this publication is: zoobank.org:pub:B89FD16E-2084-431A-ACE9-4E4362C6C3CD, and for taxonomic registration it is: urn:lsid:zoobank.org:act:66DBF909-CF1C-479C-BDA0-F324F4FFC15F. The online version of this work is archived and available from the following digital repositories: PeerJ, PubMed Central SCIE and CLOCKSS”.

## Results

More than 1,000 columnals and pluricolumnals, dozens of disarticulated ossicles from cups and arms, and 26 complete (or nearly complete) cups/calyces were collected. As a result of our investigations, the following taxa were identified: *Bactrocrinites* sp., *Codiacrinus sevastopuloi* sp. nov., *Halocrinites geminatus* (Bohatý, 2005), *Halocrinites schlotheimii* Steininger, 1831, and Flexibilia incertae sedis. Simple discoid holdfasts are also described.

### Systematic paleontology

Abbreviations used for specimen measurements include ACH, aboral cup height; ACdistW, distal width of aboral cup; ACmaxW, maximum width of aboral cup; ACproxW, proximal width of aboral cup; BConW, basal concavity width; BH, basal plate height; BW, basal plate maximum width; CrW, crown width; 1stPBH, first primibrachial height; 1stBrW, first primibrachial width; 2ndPBH, second primibrachial height; 2ndPBdistW, second primibrachial distal width; 2nd PBproxW, second primibrachial distal width, 5thPBH, fourth primibrachial height; 3rdPBW, third primibrachial width; 3rdPBH, third primibrachial height; 5thSBW, fifth primibrachial width. All measurements are in mm. Terminology for encrusting organisms follows the recommendations of Taylor & Wilson (2002).

**Table:** 

Class Crinoidea Miller, 1821
Subclass Pentacrinoidea Jaekel, 1918
Infraclass Inadunata Wachsmuth and Springer, 1885
Parvclass Cladida Moore & Laudon, 1943
Magnorder Eucladida Wright, 2017
Superorder Cyathoformes Wright et al., 2017
Superfamily Codiacrinoidea Bather, 1890
Family Codiacrinidae Bather, 1890
Subfamily Codiacrininae Bather, 1890
Genus *Codiacrinus*Schultze, 1866

#### Type species

*Codiacrinus granulatus* Schultze, 1866

#### Included species

*C. granulatus* (Schultze, 1866); *C. nicolli* Jell & Jell, 1999; *C. ornatus* (Prokop, 1973); *C. piriformis* Le Menn, 1997; *C. procerus* (Prokop, 1973); *P. rarus* (Jell & Holloway, 1983); *C. robustus* Le Menn, 1997; *C. schultzei* Follman, 1887; *C. secundus* Jell, 1999.

**Table:** 

**Figure 3:**
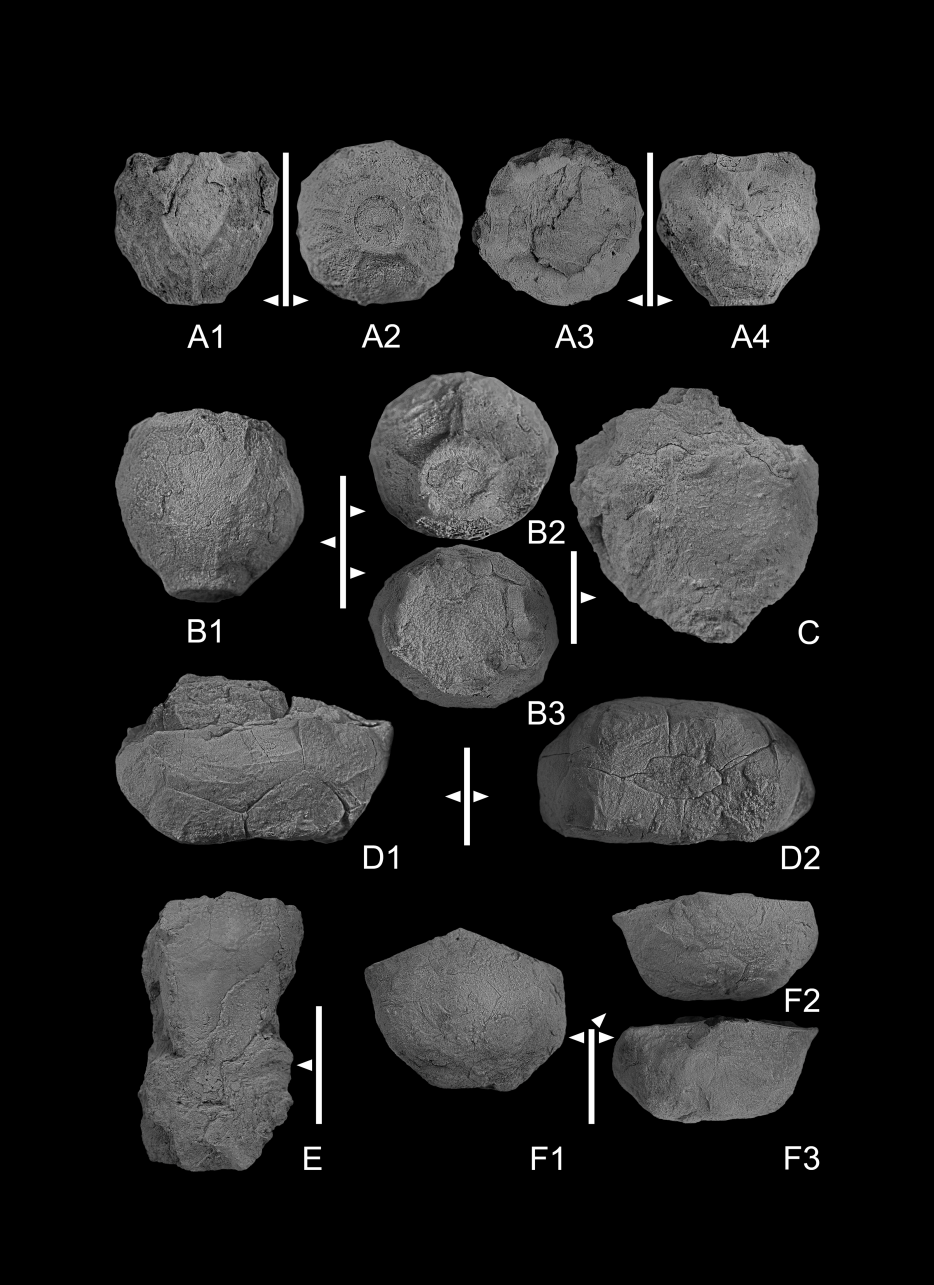
(A1–A4) *Codiacrinus sevastopuloi* sp. nov. GIUS 4-3696/Codiacrinus1, holotype; (A1) lateral view of aboral cup; (A2) basal view of aboral cup, note basal concavity bordered by ridge; (A3), oral view of aboral cup; (A4), lateral lateral view of aboral cup. (B1–B3) Codiacrinus sevastopuloi sp. nov. GIUS 4-3696/Codiacrinus2. paratype; (B1) lateral view of aboral cup; (B2) basal view of aboral cup; (B3) oral view of aboral cup. (C) lateral view of an incomplete and compressed specimen of *Codiacrinus sevastopuloi* sp. nov. GIUS 4-3696/Codiacrinus3. (D1–D2) compressed specimen of *Halocrinites* sp. GIUS 4-3696/Hsp; (D1) lateral view of aboral cup with plate boundaries visible; (D2) basal view of cup. (E) *Bactrocrinites* sp. GIUS 4- 3696/Bactrocrinites1; lateral view of incomplete aboral cup, note small radial plates and large basal plates. (F1–F3) compressed aboral cup of *Halocrinites schlotheimii*. GIUS 4- 3696/Hschloth1 (F1) oblique basal view; (F2, F3) lateral views. All specimens are from Bukowa Góra Member (Emsian), Bukowa Góra quarry, Holy Cross Mountains, southern Poland. Scale bar equals 10 mm.

*Codiacrinus sevastopuloi* sp. nov.
Figs. 3A1–3A4, 3B1–3B3, 3C

### Diagnosis

Aboral cup medium bowl shape; three or more radiating ridges from center of basal plates that project onto radial and infrabasal plates, also very fine nodose sculpturing across calyx plates; basal plates largest plates of aboral cup; radial facets ∼50% of distal width of radial plates (arms and column characters not known).

### Types

Holotype: GIUS4-3693/Codiacrinus1; paratypes: GIUS4-3693/Codiacrinus2, GIUS4-3693/Codiacrinus3.

### Occurrence

Bukowa Góra Member (Emsian), Bukowa Góra quarry, Holy Cross Mountains, southern Poland.

### Description

**Figure 4:**
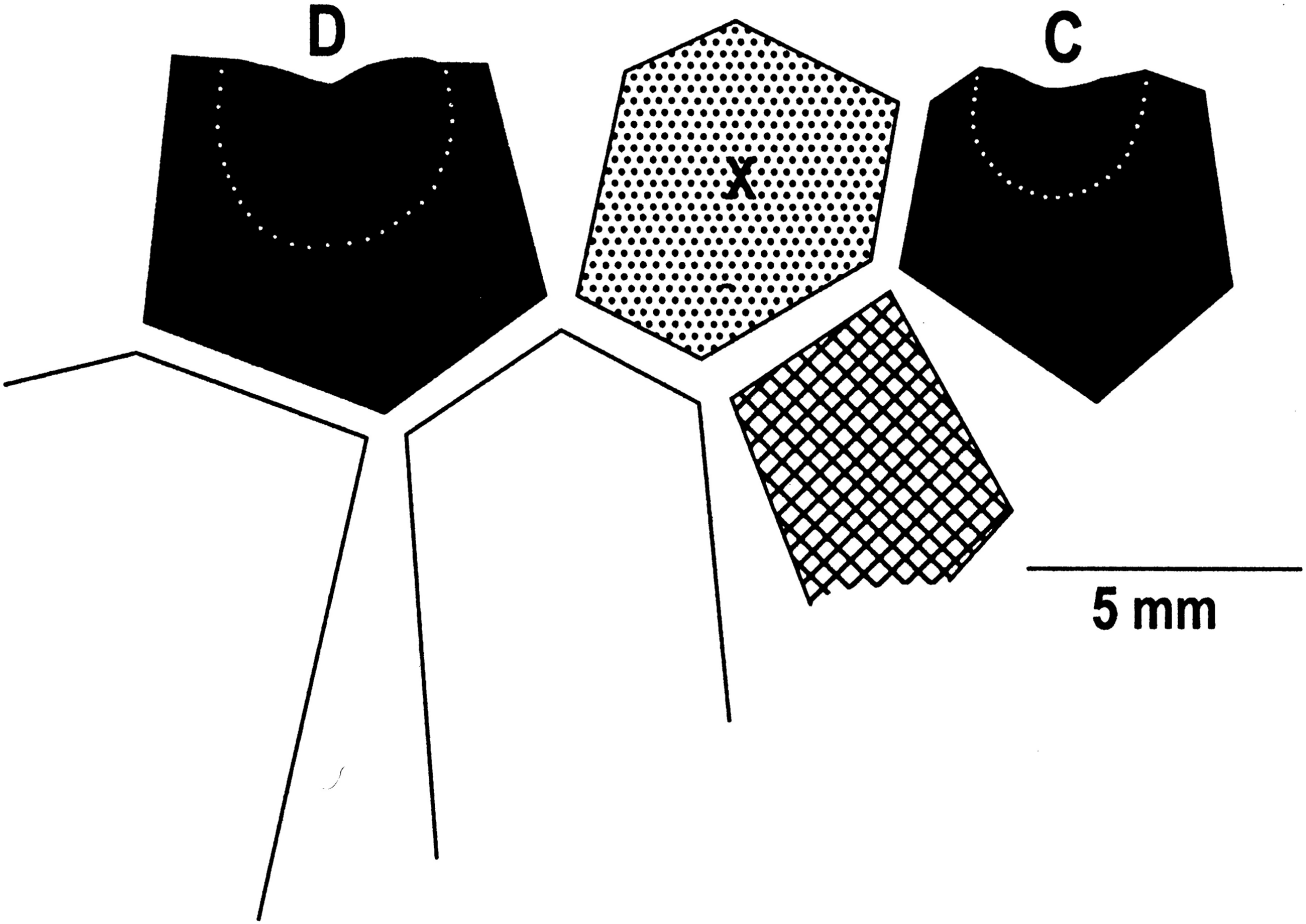
Plate drawing of the posterior of *Bactrocrinites* sp. Black pattern, radial plates; cross-hatched pattern, radianal plate; dotted pattern and X, anal X plate; dotted line, outer margin of radial facets.

Aboral cup medium globe shaped (Figs. 3A4, 3B1), height to width ratio ∼1.0, maximum width at middle aboral cup height; three or more radiating ridges from center of basal plates that project onto radial and infrabasal plates (Fig. 3A1), also very fine nodose sculpturing across calyx plates. Infrabasal circlet ∼9% of aboral cup height, extends proximally in a short neck that is truncate proximally with a shallow, circular basal concavity that occupies ∼75% of proximal aboral cup width (Figs. 4A2, 4B2). Five pentagonal infrabasal plates, ∼3.8 times wider than high, outer surface concave, sculpturing irregular nodose. Basal circlet ∼55% of aboral cup height; basal plates largest plates in aboral cup, hexagonal, ∼1.2 times higher than wide; sculpturing with radiating ridges and nodes, ridges from near the center of the plates to ridges on adjoining proximal and distal plates. Radial circlet ∼36% of aboral cup height; radial plates ∼1.2 times wider than high, pentagonal; plate sculpturing with ridges and nodes, ridges diagonal from base of radial facet to like ridges on adjoining basal plates. Radial facets angustary (∼52% of radial plate distal width), horseshoe shaped (Figs. 4A3, 4B3). Radial facets, arms, and column unknown.

### Etymology

The species name is in recognition of the substantial contributions that George D. Sevastopulo made to crinoid paleobiology, as well as paleontology and stratigraphy in general.

### Measurements

GIUS4-3693/Codiacrinus1 (holotype): ACH, 9.8; ACmaxW, 10.4; IH, 1.5; IW, 2.5; BH, 5.4; BW, 7.6; RH, 5.2; RmaxW, 6.0, RdistW, 5.3, RFW, 3.1. GIUS4-3693/Codiacrinus2 (paratype): ACH, 11.25; ACmaxW, 11.25*; IH, 1.4; IW, 5.3; BH, 8.4; BW, 6.8; RH, 5.4; RmaxW, 6.6, RdistW, 4.8, RFW, 2.5.

### Remarks

One well-preserved and two poorly preserved aboral cups are assigned to *Codiacrinus sevastopuloi* sp. nov. Both poorly preserved specimens have their shapes distorted through compaction.

Ten species of *Codiacrinus*, including *C. sevastopuloi*, are recognized herein. *C.*? *weyeri* is excluded, and it is regarded either as an aberrant individual or a member of another genus. Of these ten species, only three have arms and proximal columnals preserved (*C. robustus*, *C. schultzei*, and *C. secundus*). Thus, species diagnoses are largely based on characters of the aboral cup, which vary widely.

The most noticeable character used to differentiate species of *Codiacrinus* is the aboral cup shape, which may be low bowl, high cone, medium globe, high globe, or medium vase in shape. *Codiacrinus granulatus*, *C. nicolli*, *C. robustus*, and *Codiacrinus sevastopuloi* sp. nov. all have a medium globe-shaped aboral cup. *Codiacrinus granulatus* has a medium globe-shaped aboral cup, two poorly defined radiating ridges from the base of the radial facet onto each subjacent basal plate and perhaps some poorly developed concentric ridges, radial plates are the largest plates in the aboral cup, and the radial facets occupy ∼50% of the distal radial plate width. *Codiacrinus nicolli* has a medium globe-shaped aboral cup, very fine nodose sculpturing, radial plates are the largest plates in the aboral cup, and the radial facets occupy ∼60% of the distal radial plate width. *Codiacrinus robustus* has a medium globe-shaped aboral cup, three radiating ridges from the basal plate center and otherwise smooth sculpturing, basal plates are the largest plates in the aboral cup, and the radial facets occupy ∼50% of the distal radial plate width.

Alternatively, *Codiacrinus sevastopuloi* sp. nov. has a medium globe-shaped aboral cup, three or more radiating ridges from center of basal plates that project onto radial and infrabasal plates, also very fine nodose sculpturing across calyx plates, basal plates are the largest plates in the aboral cup, and the radial facets occupy ∼50% of the distal radial plate width.

**Table:** 

Superfamily Dendrocrinacea Wachsmuth and Springer, 1886
Family Dendrocrinidae Wachsmuth and Springer, 1886
Genus *Bactrocrinites* Schnur *in*Steininger, 1849

#### Type species

*Poteriocrinus fusiformis* Roemer, 1844.

### Included species

*Bactrocrinites birmanicus* Reed, 1908; *B. cyathus* (Schmidt, 1942); *B. depressus* (Schultze, 1866); *B. fieldi* (Springer & Slocom, 1906); *B. fusiformis* (Roemer, 1844); *B. jaekeli* (Schmidt, 1934); *B. muelleri* (Jaekel, 1895); *B*. *oklahomaensis* Strimple, 1952; *B. onondagensis* Goldring, 1954; *B. penaneachensis* Le Menn, 1985; *B. porectus* (Bohatý, 2005); *B. tenuis* (Jaekel, 1895); *B*.? *trabicus* (Schmidt, 1934); *B*. *zeileri* (Mueller in Zeiler & Wirtgen, 1855).

**Table:** 

*Bactrocrinites* sp.
Figs. 3E, 4

### Description

Relatively large aboral cup, aboral cup plates with pustulose plate sculpturing (Fig. 3E). Infrabasal plates not known. Basal plates partially preserved, inferred to be the dominant plate circlet in aboral cup (Fig. 3E). C radial plate supported beneath by radianal plate and BC basal plate; D radial plate larger that C radial plate, supported beneath by CD and DE basal plates. Radial facets large, semicircular, angustary, declivate. Two anal plates in aboral cup. Radianal presumably tetragonal, below and to the left of the C radial plate and supports the anal X plate on the upper left (Fig. 4). Anal X plate hexagonal, supported beneath by the CD basal plate and the radianal, separates and articulates with lateral sides of the C and D radial plates.

Other aspects of the aboral cup, anal sac, arms, and column are not known.

### Occurrence

Bukowa Góra Member (Emsian), Bukowa Góra quarry, Holy Cross Mountains, southern Poland.

### Remarks

Species diagnostic characters for Devonian *Bactrocrinites* include shape of the aboral cup, plate sculpturing, relative heights of aboral cup plates, and the dimensions of the infrabasal and basal plates. Unfortunately, the single specimen of *Bactrocrinites* from the Emsian of Poland is not complete (GIUS4-3693/Bactrocrinites), so aboral cup shape, relative proportions of aboral cup plates and the dimensions of the basal plates cannot be determined. The pustulose aboral cup plate sculpturing and what are inferred to be prominent (high) basal plates most closely ally this specimen with *B. fusiformis*. However, a more complete accounting of the morphology of this Emsian specimen is required before a confident species assignment can be made.

**Table:** 

Superfamily Gasterocomoidea Roemer, 1854
Family Cupressocrinitidae Roemer, 1854
Subfamily Cupressocrininae Bohatý, 2006
Genus *Halocrinites*Steininger, 1831

#### Type species

*Halocrinites schlotheimi schlotheimi* Steininger, 1831.

### Included species

*Halocrinites altus* (Schultze, 1866); *H. assimilis* (Dubatolova, 1964); *H. geminatus* (Bohatý, 2005); *H. gibber* (Bather, 1919); *H. heinorum* Bohatý & Ausich (2021); *H. inflatus inflatus* (Schultze, 1866); *H. inflatus convexus* (Hauser, 2001); *H. inflatus cuneatus* (Bohatý, 2006); *H. inflatus depressus* (Hauser, 2001); *H. minor* (Schultze, 1866); *H. nodosus* (Sandberger and Sandberger, 1856); *H. rectangularis* (Schmidt, 1941); *H. schlotheimii schlotheimii* (Steininger, 1831); *H. schlotheimii granulosus* Schultze, 1866; *H. schreueri* Bohatý, 2006; *H. tesserula* (Goldfuss, 1831); *H. townsendi* (König, 1825); and *H. urogali* Roemer, 1850.

### Remarks

**Figure 5:**
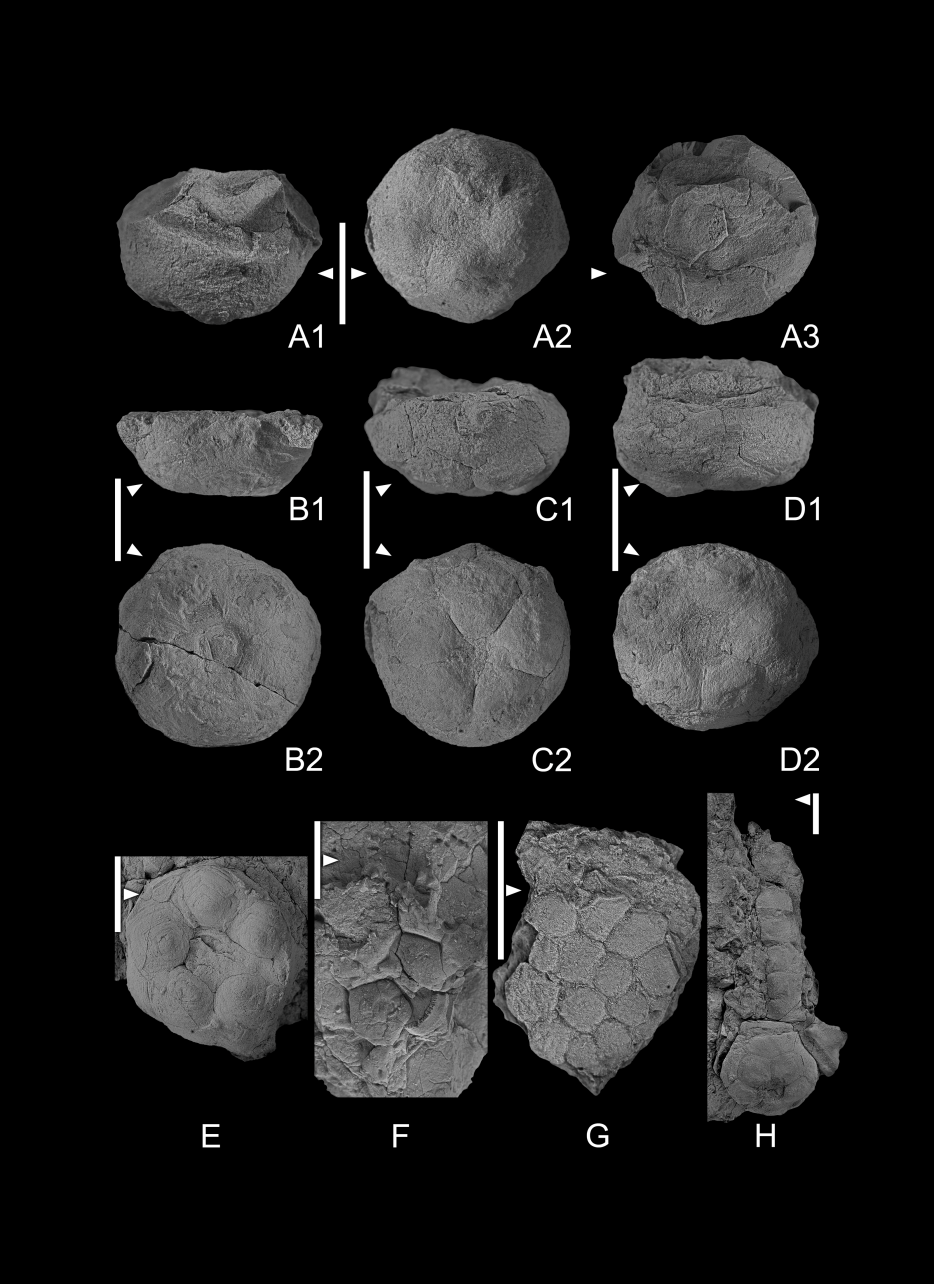
(A1–A3, B1–B2, C1–C2) *Halocrinites schlotheimii* GIUS 4-3696/Hschloth2, 5 and 4 respectively; (A1, B1, C1) lateral views of aboral cup; (A2, B2, C2) basal views of aboral cup; (A3) oral view of aboral cup. (D, H) *Halocrinites* gem GIUS 4-3696/Hgem2 and 1 respectively; (D1) lateral view of aboral cup; (D2) basal view of aboral cup. (E) *Halocrinites* with good exoplacoid sculpturing preserved; GIUS 4-3696/Hschloth6. (F, G) Crinoidea indeterminate (presumably remains of a camerate crinoid.) GIUS 4-3696/indet1 and 2 respectively. All specimens are from Bukowa Góra Member (Emsian), Bukowa Góra quarry, Holy Cross Mountains, southern Poland. Scale bar equals 10 mm.

As discussed in Bohatý & Ausich (2021), generic and specific assignments of the Cupressocrinitidae have been varied, commonly changed, and confused until recently (*e.g.*, Bohatý, 2005; Bohatý, 2006; Bohatý, 2009b; Bohatý & Herbig, 2010; Bohatý & Ausich, 2021). Two species of *Halocrinites* are recognized from the Bukowa Góra Member in Poland, including *H. geminatus* (Bohatý, 2005) and *H. schlotheimii* (Steininger, 1831). Most *Halocrinites* specimens from Poland are lacking the exoplacoid layer or are sufficiently worn that the character of the exoplacoid layer cannot be determined. One exception is specimen GIUS 4-3696Hscholth6 (Fig. 5E), although even this specimen is worn. They are differentiated on the basis of aboral cup shape, basal plate morphology, size of the infrabasal circlet relative to the size of the proximalmost columnal, and the size of the basal concavity, as described below. *Halocrinites schlotheimii* has a bowl-shaped aboral cup with a ratio of aboral cup diameter versus crown height ∼1:1.15–2.0; aboral cup typically ∼2.0 times wider than high; infrabasal plates fused into a single pentagonal plate that is confined to the basal concavity; brachials wider than high (height to width ratio ∼1:2.0–2.5); proximal columnal circular not filling entire basal concavity. In contrast, *H. geminatus* aboral cup bowl to moderately conical in shape; ratio of aboral cup diameter versus crown height ∼1:1.15–2.0; typically 2.0 times wider than high; infrabasal plates fused into a single pentalobate plate that is confined to the basal concavity; brachials wider than high (height to width ratio ∼1:2.0–2.5); proximal columnal circular not filling entire basal concavity.

Similar to the Cupressocrinitidae described by Bohatý (2009b) and Bohatý & Ausich (2021), *Halocrinites* from Poland have a variety of epizoans encrusting the outer surface of crown plates. These include trepostome bryozoans encrusted on aboral cup and brachial plates. A presumable microconchid that is attached to a radial plate, and a juvenile pelmatozoan holdfast is attached to a different radial plate. These encrustations did not induce a recognizable response from the crinoid host, so it is probable that these encrustations occurred after the death of the crinoid and, thus, are episkeletozoans (see Taylor & Wilson, 2002).

**Table:** 

*Halocrinites schlotheimii* Steininger, 1831
Figs. 3F1–F3, 5A1–A3, 5B1–B2, 5C1–C2, 53, 6A1, A2

#### Type

The type specimens for this taxon are not known.

**Figure 6:**
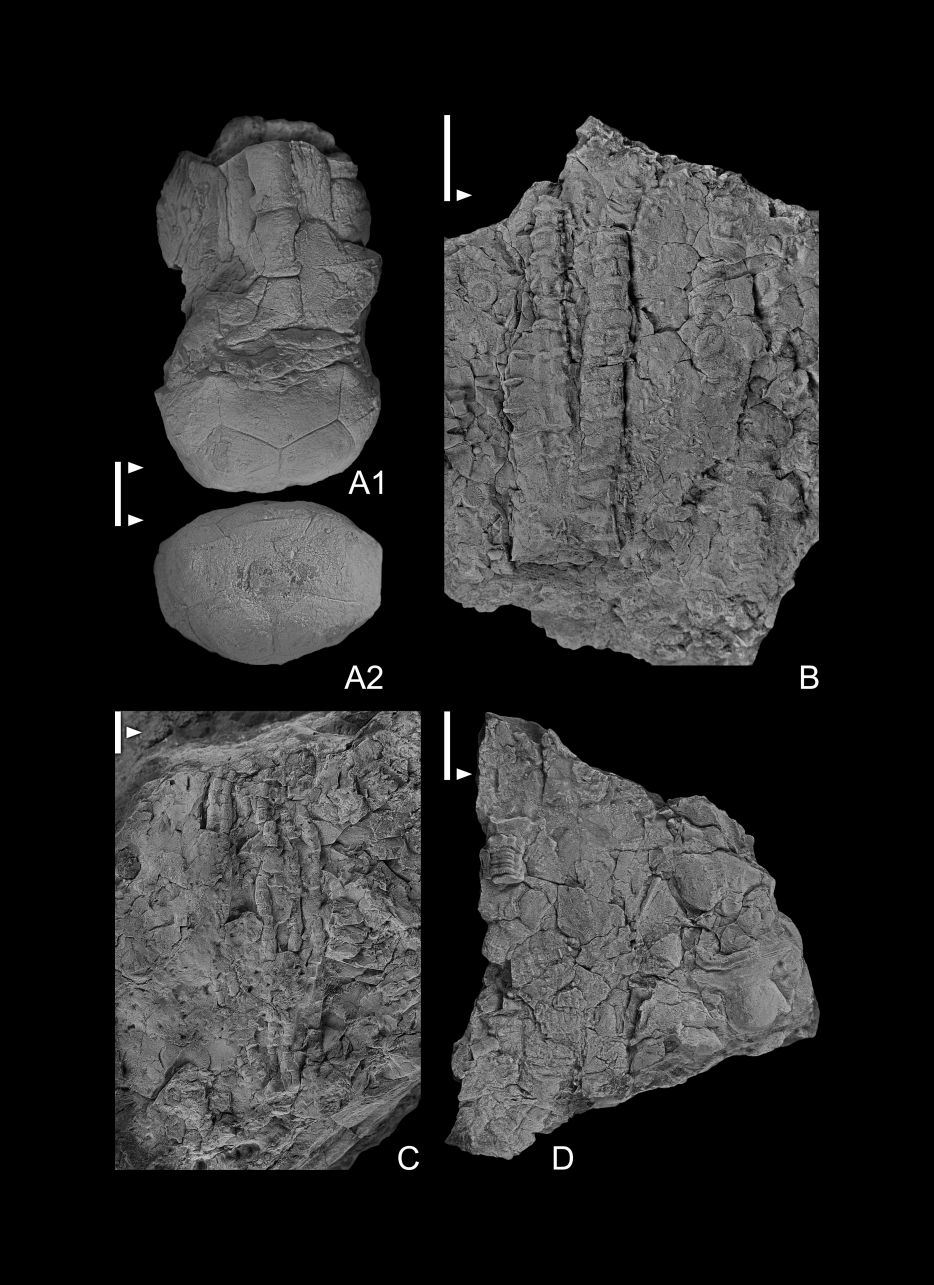
(A1–A2) *Halocrinites schlotheimii*; (A1) lateral view of partial crown, aboral cup plate boundaries distinct; (A2) basal view of a moderately compressed aboral cup. GIUS 4-3696/Hschloth3. (B–D) Crinoidea indeterminate; GIUS 4-3696/Hschloth3. (B–D) Crinoidea indeterminate; GIUS 4-3696/indet3, 4 and 5 respectively; (B, C) partial arms of an unknown cladid crinoids; (D) in upper left of specimen a pentalobate column presumably infrabasal plates attached; probably an unknown cladid). All specimens are from Bukowa Góra Member (Emsian), Bukowa Góra quarry, Holy Cross Mountains, southern Poland. Scale bar equals 10 mm.

### Diagnosis

*Halocrinites* with bowl-shaped aboral cup with a ratio of aboral cup diameter versus aboral cup height ∼1:1.15–2.0; aboral cup typically ∼2.0 times wider than high; infrabasal plates fused into a single pentagonal plate that is confined to the basal concavity; brachials wider than high (height to width ratio ∼1:2.0–2.5); proximal columnal circular not filling entire basal concavity.

### Occurrence

In Poland, *H. schlotheimii* is from the Bukowa Góra Member (Emsian), Bukowa Góra quarry, Holy Cross Mountains, Poland. Previously, this species has been described from the Eifelian and Givetian of Australia, China, Germany, Poland, and Spain (Webster & Webster, 2019).

### Description

Calyx medium sized. Aboral cup low to very low bowl shape in adults with height to maximum width ratio 0.44–0.66 (Fig. 3F); perfect pentameral symmetry; deep, subpentalobate basal concavity occupies 62–66% of proximal aboral cup width (Fig. 5E). Plates gently convex; coarse multilaminar exoplacoid sculpturing preserved on only a few specimens.

Infrabasal circlet completely in basal concavity, pentagonal. Infrabasal plates presumably five. Basal circlet ∼58–60% of aboral cup height, present on base and on vertical sides of aboral cup (Figs. 3D, 3F, 5A–5C). Five basal plates, equal in size, wider than high, smaller than radial plates. Radial circlet 40–42% of calyx height (Fig. 6A). Radial plates five, pentagonal, largest plates in aboral cup, height to width ratio 0.48–0.58. Radial facets plenary, planate; radial facet topography not known. Posterior interray plates absent from aboral cup; anal sac, if present, unknown.

Arms five, atomous, brachials uniserial; V-shaped in cross section across width of brachial plate; incompletely known (preserved only through sixth primibrachial) (Fig. 6A). First primibrachial (articular plate, see Bohatý & Ausich, 2021), very low, full width of radial facet; subsequent brachials, flat sided, equal in width to distal edge of second primibrachials; height to width ratio ∼0.65.

Proximal column narrow, attachment to base of aboral cup circular, occupies slightly more than one half of infrabasal circlet; remainder of column unknown.

### Measurements

GIUS4-3693/Hschloth3: CrH, 33.0*; ACH, 7.5; ACmaxW, 11.3; 4thPBH, 6.0; 4thPBW, 4.0. GIUS4-3693/Hschloth4: ACH, 6.3; ACdistW, 14.0; BH, 6.3; BW, 6.5; RH, 4.3; RW, 8.9.

### Remarks

Six specimens of *H. schlotheimii* are known from the Emsian of Poland (GIUS4-3693/Hschloth1 to GIUS4-3693/Hschloth6). In the collection of Polish specimens, small inidividuals tend to have more pronounced convex basal plates that nearly produce a central node.

**Table:** 

*Halocrinites geminatus* Bohatý, 2005
Figs. 5D, 5H

### Type

Holotype is SMF 75308 (see Bohatý, 2005).

### Diagnosis

*Halocrinites* with aboral cup bowl to moderately conical in shape; ratio of aboral cup diameter versus aboral cup height ∼1:1.15–2.0; typically 2.0 times wider than high; infrabasal plates fused into a single pentalobate plate that is confined to the basal concavity; brachials wider than high (height to width ratio ∼1:2.0–2.5; proximal columnal circular not filling entire basal concavity.

### Occurrence

In Poland, *H. geminatus* is from the Bukowa Góra Member (Emsian), Bukowa Góra quarry, Holy Cross Mountains. Previously, it was known from the Eifelian to early Givetian of Germany (Webster & Webster, 2019).

### Description

Calyx medium sized. Aboral cup very low bowl shape (Fig. 5D1), height to maximum width ratio 0.43; perfect pentameral symmetry; shallow, subpentalobate basal concavity occupies ∼73% of proximal aboral cup width (Fig. 5D2). Plates gently convex with multilaminar exoplacoid sculpturing (see Głuchowski, 1993a).

Infrabasal circlet completely concealed in basal concavity; outer margin of basal concavity subtetragonal, entirely covered by proximal columnal. Infrabasal plates presumably five. Basal circlet ∼56% of aboral cup height, present on base and on vertical sides of aboral cup. Five basal plates, equal in size, wider than high, much smaller than radial plates. Radial circlet ∼44% of calyx height. Radial plates five, pentagonal, largest plates in aboral cup, height to width ratio 0.60. Radial facets plenary, planate; radial facet topography not known. Posterior interray plates absent from aboral cup; anal sac, if present, unknown.

Arms five, atomous, brachials uniserial (Fig. 5H); V-shaped in cross section across width of brachial plate; incompletely known (preserved only through sixth primibrachial). First primibrachial (articular plate, see Bohatý & Ausich, 2021), very low (height to width ratio 0.16), full width of radial facet; subsequent brachials, flat sided, equal in width to distal edge of second primibrachials; height to width ratio ∼1.2.

Proximal column attachment to base of aboral cup wide, tetralobate, fills entire basal concavity covering infrabasal plates; remainder of column unknown.

### Measurements

GIUS4-3969/Hgem1: CrH, 41.0*; ACH, 9.0; ACmaxW, 21.0; ACproxW, 9.0; BConW, 6.5; BH, 7.6; BW, 7.25; RH, 5.9; RW, 12.4; 1stPbH, 1.5; 1stPbW, 13.0; 2ndPbH, 6.0; 2ndPbproxW, 13.0; 2ndPbdistW, 10.0; 3rdPBH, 5.9.0; 3rdPBW, 5.0.

### Remarks

Bohatý (2005) illustrated individuals of *H. geminatus* with a wide variety of shapes. The description above is for the Poland specimens that all have a very low bowl shape.

**Table:** 

**Figure 7:**
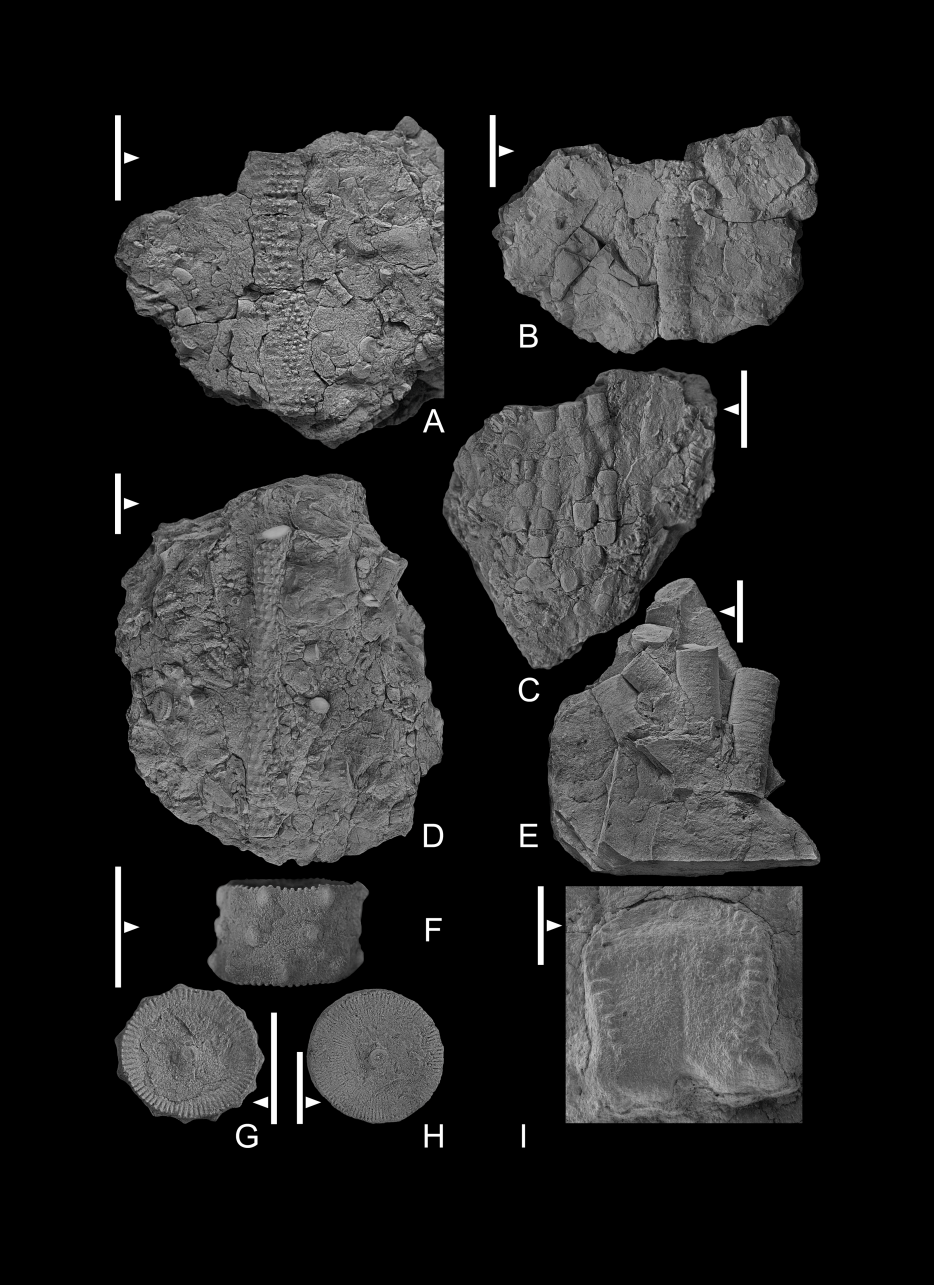
(A–H) crinoid pluricolumnals. GIUS 4-3696/indet6-13. (A, B) pluricolumnal with numerous nodes around the periphery of each columnal; (C) set of pluricolumnals; (D) pluricolumnal with nodes and perhaps some spines around the periphery of each columnal; (E) numerous pluricolumnals of a column that lacks nodes; (F) lateral view of three-columnal pluricolumnal with a few nodes around the periphery of columnals that are offset in position from one columnal to the next;(G) columnal facet with a narrow peripheral lumen and a narrow, raised perilumen; (H) columnal with a wide lumen and a narrow, raised perilumen: (I) Flexible crinoid brachial. GIUS 4-3696/flexible; note crenulate sides and on the upper margin a notch to hold a patelloid process from the next highest columnal. All specimens are from Bukowa Góra Member (Emsian), Bukowa Góra quarry, Holy Cross Mountains, southern Poland. All scale bar equals 10 mm but in case of flexible crinoid brachial it is 1 mm.

**Figure 8:**
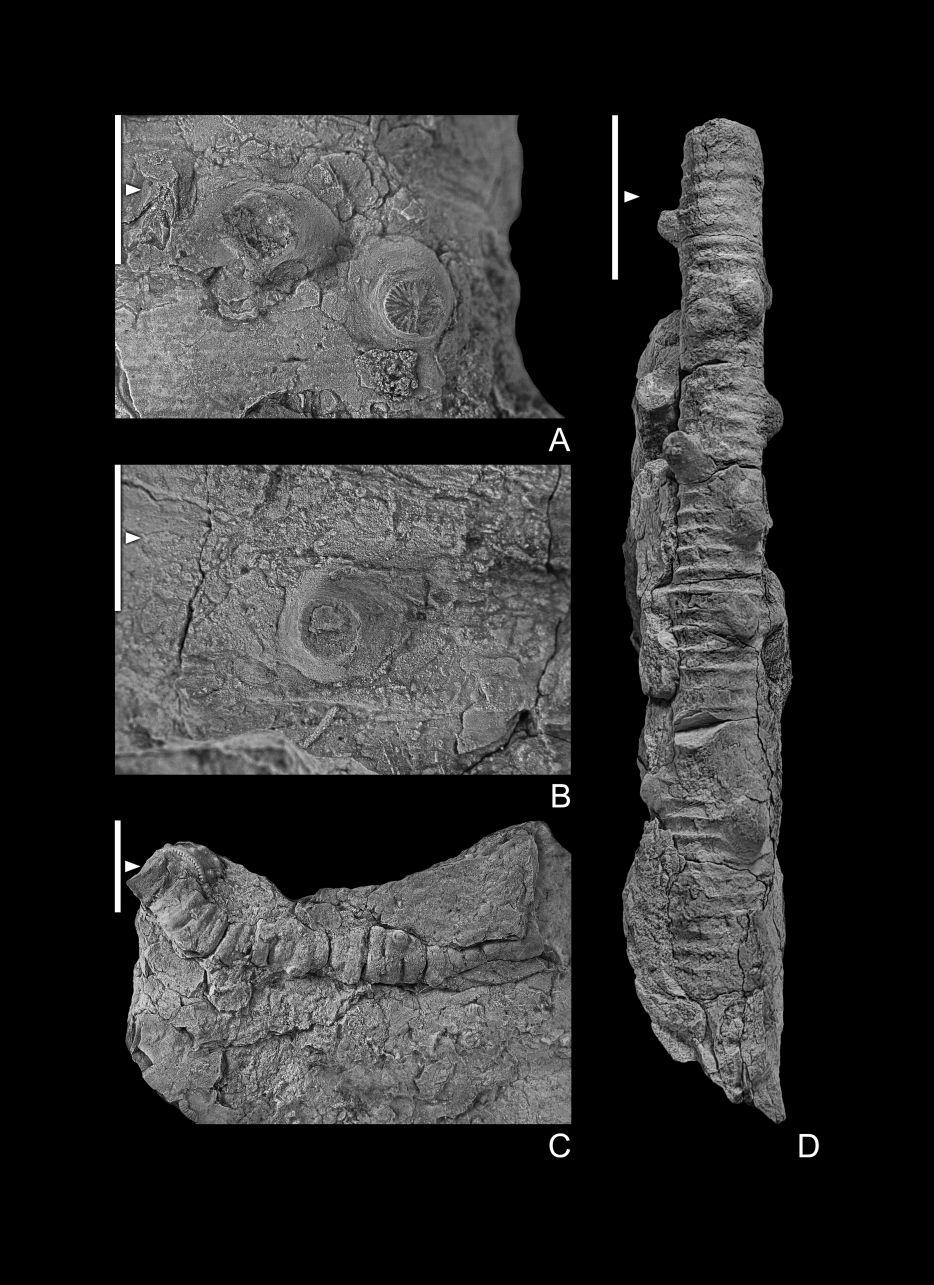
(A, B) Crinoid holdfast on stromatoporoids. GIUS 4-3696/holdfast1 and 2, respectively. (C) Crinoid pluricolumnal presumably from the dististele with broken radices; (D) pluricolumnal with elliptical columnals, presumably from a member of the Platycrinitidae; GIUS 4-3696/indet14 and 15 respectively. All specimens are from Bukowa Góra Member (Emsian), Bukowa Góra quarry, Holy Cross Mountains, southern Poland. Scale bar equals 10 mm.

Crinoidea Incertae Sedis
Figs. 5F, 5G, 6B–6D, 7A–7I, 8C, 8D,

### Occurrence

Bukowa Góra Member (Emsian), Bukowa Góra quarry, Holy Cross Mountains, southern Poland.

### Remarks

A single brachial plate is identified as a flexible crinoid (GIUS4-3693/flexible; Fig. 7I). The brachial plate is ∼4.0 times deeper than high with, only the distal facet is visible and part of the sides of the plate are visible that includes the aboral indentation where a patelloid process from the distal adjoining brachial would reside. The brachial plate is as wide as deep. A crenulated articular ridge is present along the abaxial portion of the facet, and the lateral sides of the facet are crenulated. A narrow, shallow aboral groove is present along the adaxial margin of the facet.

Poorly preserved and unidentifiable remains of several additional taxa also occur in the Bukowa Góra Member. In addition to the flexible crinoid and holdfasts mentioned below, others include camerate crinoids (Figs. 5F, 5G) and various distinctive cladid crinoids (Figs. 6B–6D). Distinctive columnals and pluricolumnals are also present (Figs. 7A, 8C, 8D). The pluricolumnals illustrated in Fig. 8C undoubtedly belong to the Platycrinitidae and may be *Platycrinites minimalis* (col.) (Głuchowski, 1993a).

**Table:** 

Simple discoid holdfasts
Figs. 8A, 8B

### Occurrence

Bukowa Góra Member (Emsian), Bukowa Góra quarry, Holy Cross Mountains, Poland.

### Remarks

Solitary rugose coral and a presumable stromatoporoid specimens associated with the described crinoids have small, discoid holdfasts cemented to their outer surface (Figs. 8A, 8B). These holdfasts are subcircular in outline and some have a slightly digitate outer margin. In one example, the holdfast articulation to the column was canted toward the long axis of a rugose coral, suggesting the crinoid was encrusted to the coral when both were alive. Therefore, these holdfasts should be considered epizoozoans (Taylor & Wilson, 2002). It is not possible to speculate on the identity of the crown that was attached to these holdfasts, and the smaller specimens may have been juveniles or from multiple radices of a single adult.

## Concluding Remarks

The first Emsian crinoids described on the basis of aboral cups and crowns are reported here from the Bukowa Góra Member in the Holy Cross Mountains of southern Poland. Named taxa include *Bactrocrinites* sp., *Codiacrinus sevastopuloi* sp. nov., *Halocrinites geminatus* (Bohatý, 2005), *Halocrinites schlotheimii* (Steininger, 1831). Taxa that can only be recognized as incertae sedis include one flexible crinoid, as many as three camerate crinoids, as many as four additional cladid crinoids, and a number of distinctive holdfasts, columnals, and pluricolumnals that cannot be matched with the crown to which they were attached. Additional collecting in the Bukowa Góra Member should yield remains of many crinoids.

Previously, *Halocrinites* (including *H. schlotheimii*) have been described from younger Devonian strata in Poland (see Fig. 1). Further, species of *Bactrocrinites*, *Codiacrinus*, and *Halocrinites* occur in other Devonian crinoid faunas from Germany and Spain (Webster & Webster, 2019).

The new crinoids reported here are from the Bukowa Góra Member of Poland (Emsian) and are an extension of the Lower to Middle Devonian crinoid faunas from across Europe, which are best represented by Emsian to Givetian crinoids from Germany and Spain (*e.g.*, Bohatý, 2005; Bohatý, 2006; Bohatý, 2009b; Bohatý & Herbig, 2010; Hauser, 2001; Hauser, 2002; Hauser, 2007). *Halocrinites* has been reported from Germany, Spain, Belgium, and Russia (Eifelian–Frasnian); wheras both *Codiacrinus* and *Bactrocrinites* have longer ranges and are cosmopolitan in distribution. In addition, to Western Europe, *Codiacrinus* is known from Gondwana terrane (northern Africa and Australia). The oldest recognized species of *Bactrocrinites* is from the middle Silurian of North America, and this genus is only known from North America and Europe. As known, *Bactrocrinites* became extinct at the Givetian–Frasnian extinction and *Bactrocrinites* and *Halocrinites* became extinct at the Frasnian-Famennian extinction.

## Additional Information and Declarations

### Data Availability

The following information was supplied regarding data availability: Codiacrinus sevastopuloi sp. nov. Holotype: GIUS4-3693/Codiacrinus1; paratypes: GIUS4-3693/Codiacrinus2, GIUS4-3693/Codiacrinus3. The specimen is deposited in the University of Silesia in Katowice, Faculty of Natural Sciences, Institute of Earth Sciences, Poland.

### New Species Registration

The following information was supplied regarding the registration of a newly described species: Publication LSID: urn:lsid:zoobank.org:pub:B89FD16E-2084-431A-ACE9-4E4362C6C3CD Codiacrinus sevastopuloi LSID: urn:lsid:zoobank.org:act:66DBF909-CF1C-479C-BDA0-F324F4FFC15F
